# SHR-A1811 (antibody-drug conjugate) in advanced HER2-mutant non-small cell lung cancer: a multicenter, open-label, phase 1/2 study

**DOI:** 10.1038/s41392-024-01897-y

**Published:** 2024-07-15

**Authors:** Ziming Li, Zhengbo Song, Wei Hong, Nong Yang, Yongsheng Wang, Hong Jian, Zibin Liang, Sheng Hu, Min Peng, Yan Yu, Yan Wang, Zicong Jiao, Kaijing Zhao, Ke Song, You Li, Wei Shi, Shun Lu

**Affiliations:** 1 Shanghai Lung Cancer Center, Shanghai Chest Hospital, Shanghai Jiaotong University School of Medicine, Shanghai, 200030 China; 2 Phase I Clinical Trial Ward, Zhejiang Cancer Hospital, Hangzhou, 310000 China; 3 Department of Thoracic Oncology, Zhejiang Cancer Hospital, Hangzhou, 310000 China; 4 Department of Lung & Gastrointestinal Oncology, Hunan Cancer Hospital, Changsha, 410031 China; 5 Thoracic Oncology Ward/Cancer Center, West China Hospital of Sichuan University, Chengdu, 610041 China; 6 Department of Thoracic Oncology, The Fifth Affiliated Hospital of Sun Yat-Sen University, Zhuhai, 519000 China; 7 Department of Medical Oncology, Hubei Cancer Hospital, Wuhan, 430000 China; 8 Department of Oncology, Renmin Hospital of Wuhan University, Wuhan, 430200 China; 9 Department of Thoracic Medicine, Harbin Medical University Cancer Hospital, Harbin, 150081 China; 10 Department of Medical Oncology, Cancer Hospital, Chinese Academy of Medical Sciences, Beijing, 100021 China; 11 Geneplus-Beijing, Co., Ltd., Beijing, 102206 China; 12 Jiangsu Hengrui Pharmaceuticals, Co., Ltd., Shanghai, 200120 China

**Keywords:** Lung cancer, Drug development

## Abstract

A dose-escalation and expansion, phase 1/2 study (ClinicalTrials.gov, NCT04818333) was conducted to assess the novel antibody-drug conjugate SHR-A1811 in pretreated HER2-altered advanced non-small cell lung cancer (NSCLC). Here, we report results from the phase 1 portion. Patients who had previously failed or were intolerant to platinum-based chemotherapy were enrolled and received SHR-A1811 intravenously at doses of 3.2 to 8.0 mg/kg every 3 weeks. Dose escalation followed a Bayesian logistic regression model that included overdose control, with subsequent selection of tolerable levels for dose expansion. Overall, 63 patients were enrolled, including 43 receiving a recommended dose for expansion of 4.8 mg/kg. All patients had *HER2*-mutant disease. Dose-limiting toxicity occurred in one patient in the 8.0 mg/kg dose cohort. Grade ≥ 3 treatment-related adverse events occurred in 29 (46.0%) patients. One patient in the 6.4 mg/kg cohort died due to interstitial lung disease. As of April 11, 2023, the 4.8 mg/kg cohort showed an objective response rate of 41.9% (95% CI 27.0–57.9), and a disease control rate of 95.3% (95% CI 84.2–99.4). The median duration of response was 13.7 months, with 13 of 18 responses ongoing. The median progression-free survival was 8.4 months (95% CI 7.1–15.0). SHR-A1811 demonstrated favourable safety and clinically meaningful efficacy in pretreated advanced *HER2*-mutant NSCLC.

## Introduction

Human epidermal growth factor receptor 2 (HER2) is a well-recognized oncogenic driver that functions across a variety of tumor types.^1–4^ HER2 alteration is associated with inferior prognosis in breast cancer, gastric cancer, and lung cancer.^5–8^ As a clinically actionable genetic abnormality, HER2-targeted monoclonal antibodies (mAbs) or tyrosine kinase inhibitors (TKIs) have substantially extended survival in patients with HER2-positive breast cancer and gastric cancer.^9–13^ Disappointingly, the advantageous effects of these targeted therapies were largely not extended to lung cancer. Currently, the non-specific platinum-based chemotherapy with or without immunotherapy remains a standard first-line treatment for advanced *HER2*-altered non-small cell lung cancer (NSCLC), based on studies in general patient populations without sensitizing molecular alterations.^14^

In NSCLC, HER2 protein overexpression was reported in 2% to 30% of cases, gene amplification in 2% to 5%, and mutation in 1% to 4% of cases.^15,16^ Accumulating evidence suggests that HER2-targeted TKIs hold potential for patients with NSCLC harboring activating *HER2* mutations, with reported objective response rates (ORRs) of up to 30% and duration of response (DoR) of up to 6.9 months.^17–22^ However, anti-HER2 mAb monotherapy had consistently demonstrated limited antitumor activity for *HER2*-altered NSCLC.^23,24^

Recently, the advent of antibody-drug conjugates (ADC), a class of therapeutic compounds designed to deliver cytotoxic agents selectively to tumor cells, has renewed hope for antibody-based therapy for *HER2*-mutant NSCLC. Trastuzumab emtansine (T-DM1) represents the first ADC to show potential clinical benefits in *HER2*-mutant NSCLC, achieving an ORR of 44% in pretreated patients; however, the median DoR was modest at 4 months.^25^ Compared with T-DM1, the novel HER2-targeted ADC trastuzumab deruxtecan (T-DXd) features a high drug-antibody ratio (DAR, 8 vs 3.5 with T-DM1) and bystander killing effect, which enables highly efficient antitumor activity not only in antigen-expressing tumor cells but also adjacent cells regardless of HER2 expression levels.^26^ T-DXd was granted accelerated approval by the US Food and Drug Administration for treating patients with previously treated *HER2-*mutant NSCLC, based on the positive findings of the DESTINY-Lung01 and 02 trials.^27,28^ At the approved dose (5.4 mg/kg), T-DXd demonstrated an ORR of 49.0%, a median DoR of 16.8 months and a median progression-free survival (PFS) of 9.9 months.

SHR-A1811 is a new ADC, comprising a humanized HER2-directed mAb (trastuzumab) conjugated to DNA topoisomerase I inhibitors (SHR169265) via cleavable tetrapeptide-based linkers.^29–31^ In pre-clinical studies, the payload of SHR-A1811 demonstrated superior membrane permeability and cytotoxicity compared with the payload of T-DXd.^29^ In addition, SHR-A1811 has a lowered DAR of 6, which provides potent bystander killing and antitumor efficacy, while potentially improving the safety profile.^29^ We conducted a phase 1/2 study to assess the safety, tolerability, pharmacokinetics, and efficacy of SHR-A1811 in patients with HER2-altered (overexpression, amplification, or mutation) NSCLC. Here, we present the results of SHR-A1811 in patients with *HER2*-mutant NSCLC from the phase 1 dose-escalation and expansion portion.

## Results

### Patient characteristics and deposition

**Fig. 1:**
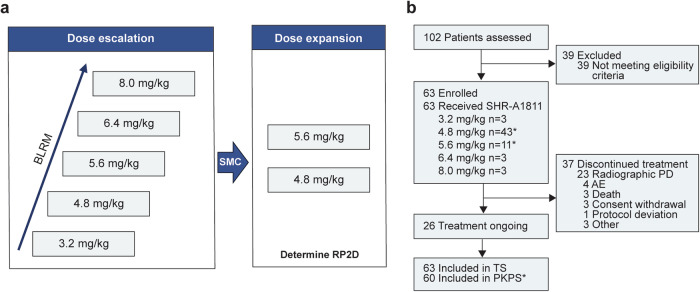
Trial profile. Study flowchart (**a**) and patient disposition (**b**) for phase 1 part of the trial are presented. TS was the primary analysis set for efficacy and safety. *Three patients (4.8 mg/kg, *n* = 2; 5.6 mg/kg, *n* = 1) with no evaluable pharmacokinetic data were excluded from PKPS. AE adverse event, BLRM Bayesian logistic regression model, PD progressive disease, PKPS pharmacokinetic parameter set, RP2D recommended phase 2 dose, SMC safety monitoring committee, TS treated set

Between May 14, 2021, and March 15, 2023, 63 patients were enrolled in the phase 1 portion of the trial (Fig. 1a). Three patients each received 3.2, 6.4, and 8.0 mg/kg of SHR-A1811, respectively, and the 4.8 and 5.6 mg/kg dose cohorts were expanded to 43 and 11 patients, respectively. As of the data cutoff (April 11, 2023), the median follow-up was 11.1 months (IQR 6.6–15.4). Twenty-six (41.3%) of the 63 patients remained on treatment, the most common reason for discontinuing SHR-A1811 was progressive disease (PD; 23 patients [36.5%]; Fig. 1b).

All patients enrolled had *HER2*-mutant disease. Additionally, two of them (3.2%) were also found to have HER2-overexpression. The patient population received a median of 2 (range 1–8) prior lines of systemic treatments, including platinum-based chemotherapy (100%), immune checkpoint inhibitors (ICIs; 61.9%) and HER2-targeted therapies (57.1%; Table 1). 27.0% of patients presented with brain metastasis at study entry.

**Table 1:** Patient demographics and clinical characteristics at baseline

	3.2 mg/kg (*n* = 3)	4.8 mg/kg (*n* = 43)	5.6 mg/kg (*n* = 11)	6.4 mg/kg (*n* = 3)	8.0 mg/kg (*n* = 3)	Total (*N* = 63)
Age (year)	58 (48–58)	56 (35–74)	59 (27–66)	63 (55–69)	67 (64–68)	58 (27–74)
*Sex*						
Male	2 (66.7)	15 (34.9)	5 (45.5)	1 (33.3)	2 (66.7)	25 (39.7)
Female	1 (33.3)	28 (65.1)	6 (54.5)	2 (66.7)	1 (33.3)	38 (60.3)
*ECOG performance status*						
0	0	2 (4.7)	5 (45.5)	0	0	7 (11.1)
1	3 (100)	41 (95.3)	6 (54.5)	3 (100)	3 (100)	56 (88.9)
*Smoking history*						
Never	3 (100)	36 (83.7)	9 (81.8)	2 (66.7)	2 (66.7)	52 (82.5)
Former	0	6 (14.0)	2 (18.2)	1 (33.3)	1 (33.3)	10 (15.9)
Current	0	1 (2.3)	0	0	0	1 (1.6)
Tumor stage IV	3 (100)	42 (97.7)	11 (100)	3 (100)	3 (100)	62 (98.4)
Adenocarcinoma	3 (100)	43 (100)	10 (90.9)	3 (100)	3 (100)	62 (98.4)
*Site of metastasis at enrollment*						
Brain	1 (33.3)	10 (23.3)	5 (45.5)	1 (33.3)	0	17 (27.0)
Liver	0	8 (18.6)	2 (18.2)	0	0	10 (15.9)
*Previous systemic treatment*						
Platinum-based chemotherapy	3 (100)	43 (100)	11 (100)	3 (100)	3 (100)	63 (100)
Immune checkpoint inhibitor	3 (100)	27 (62.8)	6 (54.5)	2 (66.7)	1 (33.3)	39 (61.9)
HER2-targeted therapy	3 (100)	26 (60.5)	4 (36.4)	0	3 (100)	36 (57.1)
Lines of previous antitumor treatment	3 (3–6)	2 (1–8)	1 (1–3)	2 (1–5)	4 (2–4)	2 (1–8)
*HER2 status*						
Over-expression^a^	0	2 (4.7)	0	0	0	2 (3.2)
Mutation	3 (100)	43 (100)	11 (100)	3 (100)	3 (100)	63 (100)
*Location of HER2 mutation*						
Kinase domain	3 (100)	40 (93.0)	11 (100)	3 (100)	3 (100)	60 (95.2)
Non-kinase domain	0	3 (7.0)	0	0	0	3 (4.8)
*Type of HER2 mutations*^b^						
A775_G776insYVMA	1 (33.3)	27 (62.8)	5 (45.5)	3 (100)	0	36 (57.1)
G776 > VC	1 (33.3)	3 (7.0)	1 (9.1)	0	0	5 (7.9)
P780_Y781insGSP	1 (33.3)	3 (7.0)	2 (18.2)	0	0	6 (9.5)
G776 > LC	0	1 (2.3)	1 (9.1)	0	0	2 (3.2)
Other^c^	0	8 (18.6)	0	0	2 (66.7)	10 (15.9)
Unknown^d^	0	1 (2.3)	2 (18.2)	0	1 (33.3)	4 (6.3)

Data are *n* (%) or median (range)

^a^Immunohistochemistry 2+ or higher (centrally assessed); HER2 expression was evaluated in 30 patients

^b^Locally assessed by next-generation sequencing or polymerase chain reaction (PCR)

^c^D769Y/V777L (*n* = 2), A771_Y772insAYVM, V777delinsGAPL, G776delinsAVGC, I655V, L755S, R966P, V659D, and V659E (*n* = 1 each)

^d^All recorded as activating exon 20 insertion, as assessed by PCR

*ECOG* Eastern Cooperative Oncology Group, *HER2* human epidermal growth factor receptor 2

### Safety and tolerability

During dose-escalation, one patient in the 8.0 mg/kg dose cohort experienced dose-limiting toxicities (DLTs) during the first treatment cycle, manifesting as grade 4 febrile neutropenia/thrombocytopenia. After the DLT assessment time window, two cases of interstitial lung disease (ILD; grade 2, *n* = 1; grade 5, *n* = 1) were reported in the 6.4 mg/kg cohort. Because ILD was a major safety concern and that DLT has been reported at the 6.4 mg/kg level in the ongoing first-in-human (FIH) trial of SHR-A1811 in advanced solid tumors,^30^ the maximum dose level selected for expansion was set below 6.4 mg/kg; additionally, given the linear pharmacokinetics at tested dose levels and the potential interpatient pharmacokinetic variability, the two higher doses of 4.8 and 5.6 mg/kg were expanded for additional evaluation.

Overall, the median duration of treatment with SHR-A1811 was 6.9 months (range, 0.7–21.2). All 63 patients had experienced at least one treatment-related adverse events (TRAEs). A summary of TRAEs occurring in ≥10% of all patients is listed in Table 2. TRAEs of grade ≥3 were reported in 29 patients (46.0%); the most frequent events included decreased neutrophil count (30.2%), decreased white blood cell count (22.2%), anemia (14.3%), and decreased platelet count (12.7%). Seven (11.1%) patients experienced treatment-related ILD (grade 1–2, *n* = 6 [9.5%]), with five in the 4.8 mg/kg cohort and two in the 6.4 mg/kg cohort. ILD of any grade occurred in 10.3% (4/39) of patients with prior ICI therapy and 12.5% (3/24) of those without; events of grade ≥3 occurred in 2.6% (1/39) and none, respectively. Serious AEs related to study drug occurred in 11 patients (17.5%), with one patient each in the 3.2 mg/kg (33.3%), 5.6 mg/kg (9.1%), and 8.0 mg/kg (33.3%) cohorts, five (11.6%) in the 4.8 mg/kg cohort, and three (100%) in the 6.4 mg/kg cohort (Supplementary Table 1). The most common treatment-related serious AEs were anemia and decreased neutrophil count (3 [4.8%] patients each). TRAEs led to dose reduction in 13 patients (20.6%), and permanent dose discontinuation in three patients (4.8%; 4.8 mg/kg, *n* = 2; 6.4 mg/kg, *n* = 1). One patient in the 6.4 mg/kg cohort died of treatment-related ILD (grade 5).

**Table 2:** Treatment-related adverse events

	3.2 mg/kg (*n* = 3)		4.8 mg/kg (*n* = 43)		5.6 mg/kg (*n* = 11)		6.4 mg/kg (*n* = 3)		8.0 mg/kg (*n* = 3)		Total (*N* = 63)	
	All grade	Grade ≥3	All grade	Grade ≥3	All grade	Grade ≥3	All grade	Grade ≥3	All grade	Grade ≥3	All grade	Grade ≥3
Any TRAE	3 (100)	1 (33.3)	43 (100)	18 (41.9)	11 (100)	5 (45.5)	3 (100)	3 (100)	3 (100)	2 (66.7)	63 (100)	29 (46.0)
Neutrophil count decreased	2 (66.7)	1 (33.3)	34 (79.1)	11 (25.6)	8 (72.7)	2 (18.2)	3 (100)	3 (100)	3 (100)	2 (66.7)	50 (79.4)	19 (30.2)
Nausea	3 (100)	0	36 (83.7)	1 (2.3)	7 (63.6)	0	2 (66.7)	0	2 (66.7)	0	50 (79.4)	1 (1.6)
White blood cell count decreased	1 (33.3)	0	32 (74.4)	9 (20.9)	7 (63.6)	2 (18.2)	3 (100)	1 (33.3)	3 (100)	2 (66.7)	46 (73.0)	14 (22.2)
Anemia	1 (33.3)	0	33 (76.7)	6 (14.0)	8 (72.7)	0	3 (100)	3 (100)	3 (100)	0	48 (76.2)	9 (14.3)
Platelet count decreased	1 (33.3)	0	21 (48.8)	5 (11.6)	0	0	3 (100)	1 (33.3)	2 (66.7)	2 (66.7)	27 (42.9)	8 (12.7)
Vomiting	3 (100)	0	24 (55.8)	1 (2.3)	7 (63.6)	0	1 (33.3)	0	3 (100)	0	38 (60.3)	1 (1.6)
Decreased appetite	0	0	23 (53.5)	0	7 (63.6)	0	2 (66.7)	0	3 (100)	0	35 (55.6)	0
Asthenia	2 (66.7)	0	12 (27.9)	2 (4.7)	4 (36.4)	0	0	0	0	0	18 (28.6)	2 (3.2)
ALT increased	1 (33.3)	0	20 (46.5)	0	5 (45.5)	0	1 (33.3)	0	0	0	27 (42.9)	0
GGT increased	1 (33.3)	0	12 (27.9)	1 (2.3)	4 (36.4)	0	1 (33.3)	0	0	0	18 (28.6)	1 (1.6)
Alopecia	0	0	20 (46.5)	0	3 (27.3)	0	2 (66.7)	0	1 (33.3)	0	26 (41.3)	0
AST increased	1 (33.3)	0	18 (41.9)	0	5 (45.5)	0	1 (33.3)	0	0	0	25 (39.7)	0
Fatigue	0	0	13 (30.2)	0	2 (18.2)	0	2 (66.7)	0	3 (100)	0	20 (31.7)	0
Constipation	0	0	12 (27.9)	0	3 (27.3)	0	2 (66.7)	0	0	0	17 (27.0)	0
Proteinuria	0	0	12 (27.9)	0	1 (9.1)	0	1 (33.3)	0	1 (33.3)	0	15 (23.8)	0
Hypoalbuminemia	0	0	11 (25.6)	0	1 (9.1)	0	2 (66.7)	0	1 (33.3)	0	15 (23.8)	0
Stomatitis	0	0	10 (23.3)	1 (2.3)	3 (27.3)	0	1 (33.3)	0	1 (33.3)	0	15 (23.8)	1 (1.6)
Hypertriglyceridemia	0	0	11 (25.6)	0	1 (9.1)	0	1 (33.3)	0	0	0	13 (20.6)	0
Hyponatremia	0	0	6 (14.0)	0	2 (18.2)	0	2 (66.7)	0	1 (33.3)	0	11 (17.5)	0
Diarrhea	0	0	8 (18.6)	0	1 (9.1)	0	0	0	2 (66.7)	0	11 (17.5)	0
Blood alkaline phosphatase increased	1 (33.3)	0	7 (16.3)	0	2 (18.2)	0	1 (33.3)	0	0	0	11 (17.5)	0
Hyperglycemia	2 (66.7)	0	7 (16.3)	0	1 (9.1)	0	0	0	0	0	10 (15.9)	0
Dizziness	0	0	8 (18.6)	0	1 (9.1)	0	0	0	1 (33.3)	0	10 (15.9)	0
Chest discomfort	0	0	7 (16.3)	0	2 (18.2)	0	1 (33.3)	0	0	0	10 (15.9)	0
Abdominal discomfort	0	0	6 (14.0)	0	0	0	1 (33.3)	0	2 (66.7)	0	9 (14.3)	0
Gastroesophageal reflux disease	0	0	7 (16.3)	0	2 (18.2)	0	0	0	0	0	9 (14.3)	0
Blood bilirubin increased	1 (33.3)	0	7 (16.3)	0	1 (9.1)	0	0	0	0	0	9 (14.3)	0
Blood LDH increased	2 (66.7)	0	5 (11.6)	0	2 (18.2)	0	0	0	0	0	9 (14.3)	0
ECG QT prolonged	0	0	7 (16.3)	0	1 (9.1)	1 (9.1)	0	0	0	0	8 (12.7)	1 (1.6)
Hyperuricemia	0	0	7 (16.3)	0	0	0	0	0	0	0	7 (11.1)	0
Interstitial lung disease	0	0	5 (11.6)	0	0	0	2 (66.7)	1 (33.3)	0	0	7 (11.1)	1 (1.6)
Bilirubin conjugated increased	1 (33.3)	0	5 (11.6)	0	0	0	1 (33.3)	0	0	0	7 (11.1)	0
Lymphocyte count decreased	0	0	4 (9.3)	1 (2.3)	2 (18.2)	2 (18.2)	0	0	0	0	6 (9.5)	3 (4.8)
Febrile neutropenia	0	0	1 (2.3)	1 (2.3)	0	0	0	0	1 (33.3)	1 (33.3)	2 (3.2)	2 (3.2)

Data are *n* (%). TRAEs of any grade occurring in ≥10% of patients, and of grade ≥3 occurring in ≥2% of patients are listed

*ALT* alanine aminotransferase, *AST* aspartate aminotransferase, *ECG* electrocardiogram, *GGT* gamma-glutamyltransferase, *LDH* lactate dehydrogenase

### Pharmacokinetics

**Fig. 2:**
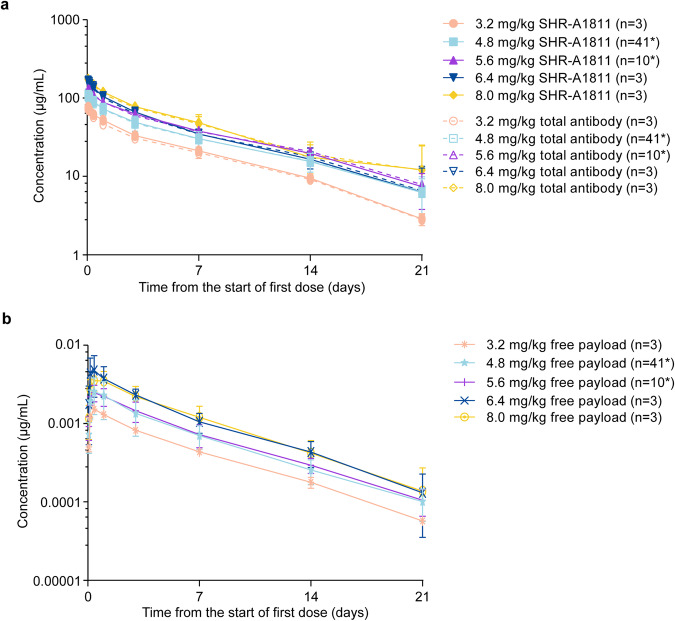
Pharmacokinetic profile of SHR-A1811 and total antibody (**a**) and free payload (**b**) after single dosing. Data are mean ± standard deviation. *Three enrolled patients (4.8 mg/kg, *n* = 2; 5.6 mg/kg, *n* = 1) had no evaluable pharmacokinetic data and were excluded from the analysis

The pharmacokinetic parameters of SHR-A1811, total antibody (TAb), and free payload after single and multiple administrations are listed in Supplementary Tables 2 and 3. After single dosing, the exposure (*C*_max_, AUC_0−21d_, AUC_0−t_, and AUC_0−∞_) of SHR-A1811, TAb, and free payload increased in a dose-dependent manner over the tested dose range (Fig. 2). The mean *t*_1/2_ of SHR-A1811 ranged from 5.1 to 7.5 days. The CL and *V*_ss_ were roughly constant across dose cohorts, demonstrating linear pharmacokinetic kinetics. The pharmacokinetic profile of TAb was similar to that of SHR-A1811 across all dose cohorts. The plasma exposure of free payload was much lower than those of ADC and TAb at all studied dose levels. Following repeated dosing, accumulation ratios of 1.2 to 1.5 were observed for AUC in both SHR-A1811 and TAb across cohorts, and the accumulation ratio of AUC in free payload ranged from 0.8 to 0.9. None of the samples from the 63 patients were tested positive for the SHR-A1811 antibody.

### Antitumor activity

**Fig. 3:**
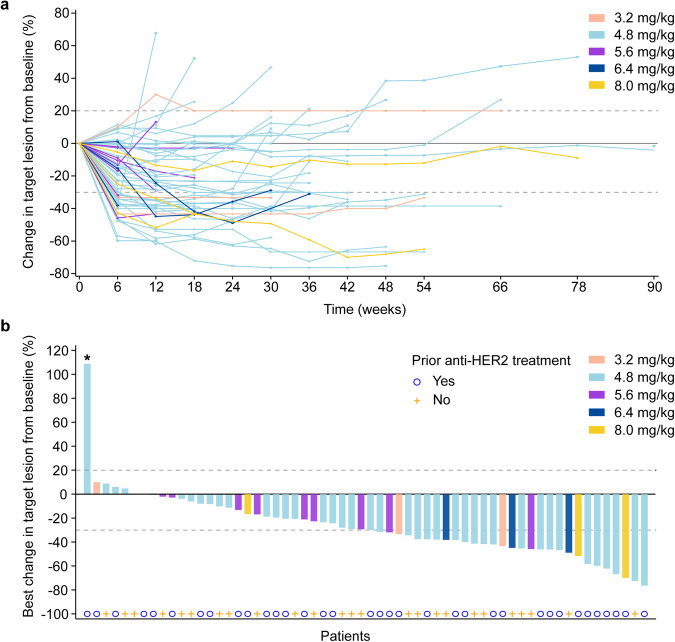
Tumor response. **a** Percentage change in target lesion over time in individual patients. **b** Best percentage change in target lesion from baseline in individual patients. * The patient was excluded from panel (**a**) as the first post-baseline tumor assessment was performed earlier than the specified time window due to clinical deterioration

Of all 63 patients, 24 patients had confirmed partial response (PR), and the ORR was 38.1% (95% CI 26.1–51.2; Fig 3). Responses with SHR-A1811 were seen in patients with (47.2%; 95% CI 30.4–64.5) or without (25.9%; 95% CI 11.1–46.3) prior anti-HER2 treatment (Fig. 3b). 33 (52.4%) patients had stable disease, and the DCR reached 90.5% (95% CI 80.4–96.4). The best overall response per cohort is presented in Table 3. Of the two expanded dose cohorts, the ORR and DCR was 41.9% (95% CI 27.0–57.9) and 95.3% (95% CI 84.2–99.4) respectively in the 4.8 mg/kg cohort, and 9.1% (95% CI 0.2–41.3) and 72.7% (95% CI 39.0–94.0) respectively in the 5.6 mg/kg cohort. With no apparent evidence of improved antitumor activity in the 5.6 mg/kg cohort, 4.8 mg/kg was determined to the recommended phase 2 dose (RP2D). Across all dose cohorts, 15 of 24 patients remained in response, and the median DoR was 10.3 months (95% CI 5.5–not reached [NR]; Fig. 3b). In the 4.8 mg/kg cohort, the median DoR was 13.7 months (95% CI 5.5–NR), with 13 of 18 responses still ongoing. As of data cutoff, PFS events occurred in 30 (47.6%) patients across all dose cohorts and in 23 (53.5%) patients in the 4.8 mg/kg cohort; the median PFS was 9.5 months (95% CI 7.1–11.7) in the overall population and 8.4 months (95% CI 7.1–15.0) in the 4.8 mg/kg cohort. One patient in the 4.8 mg/kg cohort had the longest PFS, lasting 20.5+ months. Overall survival (OS) was not mature with a total of 14 (22.2%) events recorded.

**Table 3:** Tumor responses

	3.2 mg/kg (*n* = 3)	4.8 mg/kg (*n* = 43)	5.6 mg/kg (*n* = 11)	6.4 mg/kg (*n* = 3)	8.0 mg/kg (*n* = 3)	Total (*N* = 63)
Best overall response, *n* (%)						
Complete response	0	0	0	0	0	0
Partial response	2 (66.7)	18 (41.9)	1 (9.1)	1 (33.3)	2 (66.7)	24 (38.1)
Stable disease	1 (33.3)	23 (53.5)	7 (63.6)	1 (33.3)	1 (33.3)	33 (52.4)
Progressive disease	0	1 (2.3)	1 (9.1)	0	0	2 (3.2)
Not evaluable	0	1 (2.3)	2 (18.2)	1 (33.3)	0	4 (6.3)
ORR, % (95% CI)	66.7 (9.4–99.2)	41.9 (27.0–57.9)	9.1 (0.2–41.3)	33.3 (0.8–90.6)	66.7 (9.4–99.2)	38.1 (26.1–51.2)
DCR, % (95% CI)	100 (29.2–100.0)	95.3 (84.2–99.4)	72.7 (39.0–94.0)	66.7 (9.4–99.2)	100.0 (29.2–100.0)	90.5 (80.4–96.4)
PFS (months), median (95% CI)	11.7 (6.7–NR)	8.4 (7.1–15.0)	NR (1.4-NR)	NR (1.8–NR)	11.2 (4.3–NR)	9.5 (7.1–11.7)
DoR (months), median (95% CI)	7.9 (5.4–NR)	13.7 (5.5–NR)	NR (NR)	NR (NR)	5.5 (2.6–NR)	10.3 (5.45–NR)

*CI* confidence interval, *DCR* disease control rate, *DoR* duration of response, *ORR* objective response rate, *PFS* progression-free survival, *NR* not reached

The predominate *HER2* mutation subtype was exon 20 insertions (88.9%). Other mutations included single-nucleotide variants in exons 17, 19 or 20 (Supplementary Table 4). HER2 immunohistochemistry (IHC) 2+ and IHC 3+ was found in two of 30 evaluated patients. Tumor response with SHR-A1811 was seen regardless of *HER2* mutation subtypes or detectable HER2 expression (Supplementary Tables 4 and 5).

**Fig. 4:**
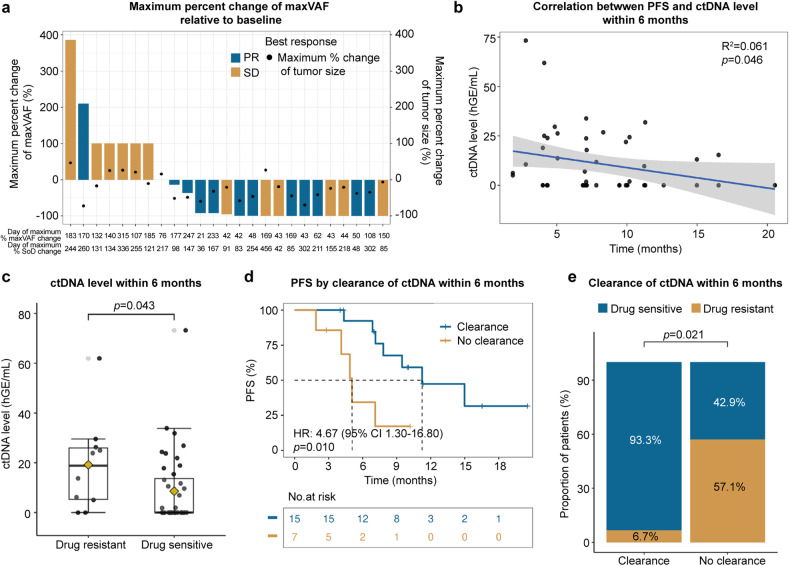
Biomarker analysis of ctDNA level within 6 months of treatment. **a** Maximum percent change in VAF and SoD during dosing relative to baseline. 25 patients (best response, PR, *n* = 12; SD, *n* = 13) with blood samples collected at both baseline and during dosing were included. **b** Linear correlation between ctDNA level within 6 months of treatment initiation and PFS. **c** Distributional differences in ctDNA levels between drug-resistant and drug-sensitive patients within 6 months of drug initiation. **d** Kaplan–Meier plot of PFS according to ctDNA clearance within 6 months. **e** Proportion of drug-resistant versus drug-sensitive patients according to ctDNA clearance within 6 months. Twenty-two patients (≥2 blood samples collected, or 1 sample collected due to recurrence or death within 6 months) were included in the analyses. *PFS* progression-free survival, *PR* partial response; *SD* stable disease; *SoD* sum of diameter, *VAF* variant allele frequency

Among the subset of patients evaluable for ctDNA, a significant linear correlation was found between the maximum change in variant allele frequency (VAF) and the sum of tumor diameters (*p* = 0.016; Supplementary Fig. 1a), with 11 of 12 (93.3%) of patients with the best efficacy assessment of PR showing a reduced VAF relative to baseline (Fig. 4a). Early clearance of ctDNA within 6 weeks was not predictive of treatment efficacy (Supplementary Fig. 1b-e). However, extending the monitoring period to 6 months revealed a significant linear correlation between the overall ctDNA level and PFS time (*p* = 0.046; Fig. 4b). ctDNA levels were also significantly higher in drug-resistant patients versus drug-sensitive patients (*p* = 0.043; Fig. 4c). Patients achieving ctDNA clearance within 6 months had extended PFS (HR 4.67, 95% CI 1.30–16.80; *p* = 0.010; Fig. 4d) and were less likely to develop drug resistance (*p* = 0.021; Fig. 4e).

## Discussion

Currently, *HER2*-mutant NSCLC are mainly managed with chemotherapy, immunotherapy and TKIs, with varying clinical outcomes, and there remains an unmet clinical need. In this study, we investigated ADC therapy in heavily pretreated *HER2*-mutant NSCLC. The results showed manageable toxicities and encouraging antitumor activity with SHR-A1811 for *HER2*-mutant NSCLC, with no new safety signals observed.

With a comparable median treatment duration, the frequency and spectrum of TRAEs with SHR-A1811 appeared generally in line with those documented for T-DXd in advanced NSCLC.^27,28,32^ Nevertheless, this preliminary observation was based on a moderate sample size, and warrants confirmation in phase 2 part of this trial and in larger-scale studies of SHR-A1811. DLT was reported in only one patient in the 8.0 mg/kg dose cohort. Treatment-related serious AEs were reported in 17.5% of patients, with hematological AEs being the most prevalent; these events could be effectively managed with standard supportive care. The rate of TRAEs leading to treatment discontinuation was low with SHR-A1811 in advanced NSCLC (4.8%), aligning with previous report of SHR-A1811 in a pan-tumor setting (2.6%).^30,31^ This favorable tolerability observed for SHR-A1811 is attributed to its unique design features. A chiral cyclopropyl group has been introduced between the linker and toxin, which enhances chemical stability and prevents AEs due to premature toxin release. Additionally, the highly potent toxin used in SHR-A1811 allows for a lower DAR of 6, potentially reducing circulating toxin levels.^29^ Unlike most other HER2-directed ADCs in clinical development for NSCLC, which utilize microtubule inhibitors as payloads,^33^ SHR-A1811 employs a topoisomerase I inhibitor. This approach may mitigate the risk of neuropathy associated with microtubule inhibitors.^34^ ILD is a significant risk for NSCLC patients treated with DXd-based ADCs.^27,28,35^ At the approved dose of 5.4 mg/kg for pretreated *HER2*-mutant NSCLC, the overall incidence of treatment-related ILD with T-DXd was 12.9% (grade ≥3, 2%; grade 5, *n* = 1).^28^ In the present study, 11.1% of 63 patients experienced ILD, with all cases being grade 1–2, except for one grade 5 case in the 6.4 mg/kg cohort. At the RP2D of 4.8 mg/kg, 11.6% of 43 patients reported low-grade ILD, supporting a relatively low risk. In our study, no association was observed between the onset of ILD and prior ICI exposure. Nevertheless, this analysis was limited by a small sample size and the relationship will be further explored in the subsequent phase 2 study of SHR-A1811. To date, the exact mechanism of action of ADC-induced ILD is not fully understood, and risk factors associated with ILD remain to be identified. Prophylactic management, patient education on self-monitoring, and early recognition of symptoms are important for minimizing the pulmonary toxicity of ADCs in future studies.

The pharmacokinetic profile of SHR-A1811 was in consistence with T-DXd or other DXd-based ADCs.^36,37^ SHR-A1811 increased dose-dependently at 3.2 to 8.0 mg/kg. The pharmacokinetic profile of SHR-A1811 and TAb were consistent. Notably, the plasma concentration of free payload was low, implying that the linker of SHR-A1811 was stable in plasma, which spared the normal tissues from cytotoxicity. The pharmacokinetic results were echoed by the favorable safety profile.

HER2 was a well clinically validated activating oncogenic driver in NSCLC, however, treatments targeting HER2 lead to mixed results.^17–21^ No HER2-targeted therapies were approved for NSCLC until the advent of T-DXd. In the DESTINY-Lung01 trial, T-DXd at 6.4 mg/kg yielded an ORR of 55%, a median DoR of 9.3 months, and a median PFS of 8.2 months in pretreated *HER2*-mutant NSCLC.^27^ In the subsequent DESTINY-Lung02 trial, T-DXd at 6.4 mg/kg and the approved dose of 5.4 mg/kg showed ORRs of 56.0% and 49.0%, and DoRs of not reached and 16.8 months respectively for *HER2*-mutant NSCLC.^27,28^ SHR-A1811 at the RP2D of 4.8 mg/kg showed generally similar efficacy with T-DXd at 5.4 mg/kg in *HER2*-mutant NSCLC, with an ORR of 41.9%, a durable DoR of 13.7 months (not mature), and PFS of 8.4 months. Nevertheless, given the difference in study design and patient characteristics, between-study comparisons should be interpreted cautiously. Notably, a high proportion of the responders to SHR-A1811 had prior anti-HER2 treatment, suggesting the presence of alternative resistance mechanisms to anti-HER2 ADC (compared with anti-HER2 TKI), which warrants further investigation. The efficacy of SHR-A1811 in *HER2*-mutant NSCLC was also seen across different *HER2* mutation subtypes. Evidence of activity of anti-HER2 agents in tumors harboring *HER2* exon 19 mutation, an uncommon mutation type, has been especially limited.^25^ Our results corroborate with findings with T-DXd, and support a role of HER2-directed ADC in treating patients with exon 19 substitutions. Notably, a higher dose of SHR-A1811 at 5.6 mg/kg did not yield an improvement in tumor response compared to the response at 4.8 mg/kg. The exact reason was unclear, but the limited sample size and peak of the COVID-19 pandemic during the enrollment for the higher dose cohort may have influenced the outcome and complicated data interpretation. Given the promising efficacy and favorable tolerability of SHR-A1811 at 4.8 mg/kg demonstrated in this study and the preceding FIH study in advanced solid tumors,^30,31^ and no signal of improved efficacy observed with 5.6 mg/kg compared to 4.8 mg/kg in both studies, further evaluation of the 5.6 mg/kg dose level with additional patients was not undertaken after the minimum required sample size for dose expansion was met.

ctDNA clearance and VAF have been reported to be associated with tumor burden and are predictive of long-term survival outcomes with TKI and ICI treatment in multiple cancers.^38–40^ Further, it is assumed that molecular response as measured by percentage change in maximum VAF during the dosing period relative to baseline may correspond with radiographic changes (e.g. best percent change in sum of diameter from baseline).^40,41^ A combination of ctDNA measurement with radiographic assessments may improve tumor burden evaluation and refine the stratification of patient prognosis. In this study, maximum percent change in VAF relative to baseline was associated with change of tumor size, and improved PFS was observed in patients who achieved clearance of ctDNA within 6 months. Although early clearance of ctDNA within 6 weeks was previously shown to be predictive of efficacy of TKI, ICI, and HER3 ADC in lung cancer,^35^ no clear correlation was observed in this study. This discrepancy may stem from differences in tumor characteristics with *HER2*-mutant disease or a limited sample size.

This study was designed to evaluate SHR-A1811 in HER2-overexpressing, -amplified, or -mutant NSCLC. However, the study population predominantly had *HER2*-mutant NSCLC. De novo HER2 overexpression and amplification are detected more often in smokers.^42,43^ The low prevalence of smokers in Chinese female NSCLC patients may lead to the under representation of these alterations. Alternatively, the predominance of *HER2* mutant disease aligns with emerging external evidence indicating the superior efficacy of anti-HER2 ADC (T-DXd) in treating *HER2*-mutant advanced NSCLC during the patient recruitment period for the study. This may reflect the critical role of benefit-to-risk assessment for patients in decision-making on clinical trial participation.

The study has several limitations. Firstly, intrinsic to the exploratory nature of phase 1 clinical trials, the sample size was relatively small. In particular, the biomarker analysis included a small subset of study population, which limited data interpretation. Secondly, assessments of *HER2* alternations for study entry were based on heterogenous local testing. Thirdly, CNS surveillance has not been systematically performed in all patients. Emerging evidence from the DESTINY-Lung01/02 trials suggests intracranial activity of HER2-directed ADC in *HER2*-mutant NSCLC.^44^ The intracranial efficacy of SHR-A1811 requires further research. Additionally, follow-up period is relatively short. Long-term data are being collected, and will be presented in a subsequent publication.

In summary, SHR-A1811 showed manageable safety profile. SHR-A1811 at a dose of 4.8 mg/kg demonstrated promising antitumor activity in patients with pretreated *HER2*-mutant NSCLC.

## Materials and methods

### Study design and patients

This was a multicenter, single-arm, open-label, dose-escalation, dose-expansion, phase 1/2 trial (NCT04818333). This study adhered to the Declaration of Helsinki, the International Council for Harmonization Good Clinical Practice, and local regulations. The trial protocol was reviewed and approved by the independent ethics committees of the leading clinical site (Shanghai Chest Hospital, #LS2115) and each participating hospital. All patients provided written informed consent prior to study enrollment.

In the phase 1 portion of this study, eligible patients had advanced or metastatic HER2-overexpressing (IHC 2+ or higher), HER2-amplified (HER2/CEP17 ≥ 2 by fluorescence in situ hybridization [FISH] or gene copy number ≥5 by next generation sequencing [NGS]^45^), or activating *HER2*-mutant (by next-generation sequencing [NGS] or polymerase chain reaction [PCR]; blood-based test allowed during the dose-escalation phase) NSCLC, according to local assessment. Patients were also required to have experienced treatment failure with platinum-based chemotherapy for advanced or metastatic disease or were intolerant to chemotherapy; had disease recurrence or metastases within 6 months of platinum-based neoadjuvant or adjuvant therapy, or radical chemoradiotherapy for locally advanced disease; or had disease progression during or after treatment for recurrence or metastases occurred ≥6 months after platinum-based neoadjuvant, adjuvant, or radical chemoradiotherapy for locally advanced disease. Other inclusion criteria included age of 18–75 years, Eastern Cooperative Oncology Group (ECOG) performance status of 0/1, ≥1 measurable lesion according to Response Evaluation Criteria in Solid Tumors (RECIST) version 1.1; and adequate organ functions. Submission of tumor tissue was optional in the phase 1 part of trial; if tissue was available, HER2 expression was retrospectively analyzed in a central lab as part of the biomarker analysis. Patients were not eligible if any of the following conditions were identified: untreated or active central nervous system metastases, systemic antitumor treatment within 4 weeks before study initiation, previous anti-HER2 ADC treatments, other concurrent oncogenic driver genes which had approved targeted drugs, secondary *HER2* mutation or amplification occurred after acquired resistance to EGFR-TKIs. Additional details are provided in the protocol.

### Treatment and assessments

Eligible patients were enrolled to receive escalating doses of 3.2, 4.8, 5.6, 6.4, and 8.0 mg/kg of SHR-A1811 intravenously every 3 weeks. The starting dose of 3.2 mg/kg was based on the favorable tolerability and preliminary clinical activity observed with SHR-A1811 in the FIH trial in patients with *HER2*-expressing/mutated advanced solid tumors (doses of 1.0–8.0 mg/kg every 3 weeks were assessed);^31^ of the six patients treated at 3.2 mg/kg, only one experienced a grade ≥3 TRAE and none reported ILD, serious TRAEs, or TRAEs leading to treatment discontinuation. Dose escalation was implemented according to a 2-parameter Bayesian logistic regression model (BLRM), which included the overdose control (EWOC) criteria,^46,47^ together with considerations of safety and pharmacokinetics. A minimum of three patients were enrolled per dose level. The BLRM estimated and updated the probabilities of a patient experiencing a DLT (see Supplementary file for definition) in the first cycle at each dose level sequentially. The overdose control was set at a <30% posterior probability of the DLT rate falling within the interval suggesting excessive toxicity (ie. [0.33, 1]). After all patients completed evaluation of DLTs in the dose-escalation phase, the safety monitoring committee chose the doses for expansion on the basis of a thorough review of the safety, pharmacokinetics, and antitumor activity. The dosing schedule was the same as for the escalation phase. Patients received SHR-A1811 until PD, intolerable toxicity, patient withdrawal, or investigator decision. Treatment beyond progression was permitted as long as the patients could still derive benefit as judged by the investigator.

Safety was evaluated until 90 days after administration of the last dose, with AEs graded per National Cancer Institute Common Terminology for Adverse Events version 5.0. Tumor assessments according to RECIST version 1.1 were performed by the investigator at baseline, every 6 weeks, and then every 12 weeks since week 54 until PD, start of new antitumor therapy, loss of follow-up, or death. For CNS surveillance, assessment was performed only for known or suspected lesions, if clinically indicated, or at the discretion of the investigator. If patients discontinued SHR-A1811 for reasons other than PD, imaging assessment continued on the original schedule until either PD or start of new antitumor therapy. All complete responses or PRs required confirmation ≥4 weeks after the initial response was noted.

Blood samples for pharmacokinetic analyses were collected pre-dose, within 5 min, 2, 8, 24, 72 h, 7, 14 day after the end of infusion during the first cycle; pre-dose and within 5 min post-dose on day 1 of cycle 2; pre-dose, within 5 min, 2 h, 7, and 14 day post-dose during cycle 3; pre-dose and post-dose on day 1 of cycles 4, 6, and 8; and pre-dose every 3 cycles since cycle 11. Blood samples for immunogenicity analyses were collected pre-dose on day 1 of cycle 1, 2, 3, 4, 6 and 8; from cycle 11 onwards, samples were collected pre-dose on day 1 of every 3 cycles.

For exploratory biomarker analysis, ctDNA was evaluated with blood samples collected at baseline, and on the first day of each treatment cycle in a subset of 28 consecutive patients from a single center (Supplementary Table 6). NGS was performed on 107 blood samples with a panel of 1021 cancer-related genes. The DNA library construction and sequencing were supported by the Geneplus-Beijing Institute (Beijing, China) using a DNBSEQ-T7RS sequencer (MGI Tech). See supplementary methods for details on NGS. Additionally, treatment efficacy was analyzed according to *HER2* mutation subtype (NGS or PCR on blood or tumor tissue; *n* = 63) and HER2 expression (immunohistochemistry on available tumor tissue; *n* = 30).

### Endpoints

The primary endpoints were maximum tolerated dose, RP2D, and safety. The secondary endpoints included pharmacokinetic parameters, immunogenicity, and antitumor activity. Pharmacokinetic parameters included peak concentration (*C*_max_), time to *C*_max_ (*T*_max_), area under the time-concentration curve (AUC) from time zero to the last measurable concentration (AUC_0−t_), AUC from time 0 extrapolated to infinity (AUC_0−∞_), AUC from time 0 extrapolated to day 21 (AUC_0−21d_), terminal elimination half-life (*t*_1/2_), mean residence time (MRT), clearance (CL), volume of distribution at steady state (*V*_ss_), and accumulation ratio (*R*_ac_) for SHR-A1811, TAb (comprising conjugated and unconjugated antibody), and free payload. Efficacy endpoints included ORR, DCR, DoR, PFS and OS.

### Statistical analyses

No formal power calculation was done. In the dose-escalation part, the number of patients was dependent on the occurrence of DLTs and the nature of the dose-escalation design. In the dose-expansion part, approximately eight to 30 patients per selected dose cohort were anticipated to be sufficient for the study objectives.

Safety and efficacy were analyzed in the treated set, which included all patients who received ≥1 dose of SHR-A1811. Pharmacokinetic parameters were assessed in patients who received ≥1 dose of SHR-A1811 and had ≥1 evaluable pharmacokinetic parameter. Pharmacokinetic parameters were estimated by non-compartmental method using Phoenix WinNonlin software (version 8.3; A Certara Company, USA). The 95% CIs of ORR and DCR were calculated with the use of Clopper Pearson method. Median PFS and DoR were estimated using the Kaplan-Meier method, with the corresponding 95% CIs calculated using the Brookmeyer-Crowley methods with log-log transformation. For biomarker analysis, Pearson correlation was used to assess the linear association between ctDNA levels and PFS time; the rank-sum test compared ctDNA levels between drug-resistant and sensitive patients; Fisher’s exact test evaluated the proportion of drug-resistant and sensitive patients with ctDNA clearance within 6 months; and the Log-rank test compared PFS outcomes between patients with and without ctDNA clearance within 6 months. Statistical analyses were conducted using SAS (version 9.4) or R (version 4.2.0).

### Supplementary information

Supplementary files

## Data Availability

Researchers can request access to deidentified patient-level data 24 months after study completion. Proposals outlining the reasons for the request should be sent to the corresponding author (shunlu@sjtu.edu.cn). The leading clinical site and sponsor will review for intellectual property and confidentiality considerations. A data access agreement must be signed before accessing the requested data.
