# Supplementary material for: SHR-A1811 (antibody-drug conjugate) in advanced HER2-mutant non-small cell lung cancer: a multicenter, open-label, phase 1/2 study
Source: Signal Transduct Target Ther. 2024 Jul 15;9:182. doi: 10.1038/s41392-024-01897-y (PMC11247081; doi:10.1038/s41392-024-01897-y)
Supplement: Supplementary file 1 — Supplementary files [file 41392_2024_1897_MOESM1_ESM.docx]

Supplementary Materials for

**SHR-A1811 (antibody-drug conjugate) in advanced HER2-mutant non-small cell lung cancer: a multicenter, open-label, phase 1/2 study**

Ziming Li, Zhengbo Song, Wei Hong, Nong Yang, Yongsheng Wang, Hong Jian, Zibin Liang, Sheng Hu, Min Peng, Yan Yu, Yan Wang, Zicong Jiao, Kaijing Zhao, Ke Song, You Li, Wei Shi, Shun Lu

Correspondence to: shunlu@sjtu.edu.cn

**This PDF file includes:**

Materials and Methods

Figures. S1

Tables S1 to S6

## Materials and Methods

## Definition of dose-limiting toxicities (DLTs)

DLT was defined as any AE related to study treatment deemed by the investigator: hematologic toxicities including grade 4 neutropenia lasting ≥7 days with or without granulocyte colony-stimulating factor, grade ≥3 febrile neutropenia, grade 4 thrombocytopenia or grade 3 thrombocytopenia with clinically meaningful bleeding, and grade 4 anemia; hepatotoxicity including grade 4 increased alanine aminotransferase (ALT) or increased aspartate aminotransferase (AST), grade 3 ALT or AST with grade ≥2 increased blood bilirubin, and grade 3 ALT or AST lasting ≥3 days; and grade ≥3 non-hematologic toxicity with the specific exception of transient or asymptomatic single laboratory test abnormalities, grade ≥3 nausea, vomiting, diarrhea, or anorexia could not restored to grade ≤2 with symptomatic treatment for ≥3 days, grade ≥3 fatigue could not be controlled to grade ≤2 after symptomatic treatment lasting >7 days, and symptomatic cardiac failure congestive.

Next generation sequencing

Cell-free DNA (cfDNA) and germline genomic DNA (gDNA) were extracted from plasma and leukocytes using the QIAamp Circulating Nucleic Acid Kit and the QIAamp DNA Blood Mini Kit (Qiagen, Hilden, Germany), respectively, cfDNA with ~170 bp and gDNA with > 500 bp were retained for subsequent processing. Indexed libraries for NGS were constructed using gDNA sheared into 200-250 bp fragments, and the cfDNA was processed for end-repairing and A-tailing reactions, and targeted adapters ligation. After PCR amplification, all libraries were hybridized to a custom-designed 1021 cancer-related gene panel. The DNA library construction and sequencing were supported by the Geneplus-Beijing Institute (Beijing, China) using a DNBSEQ-T7RS sequencer (MGI Tech). GATK was used to de-duplicate the reads, local realignment and base quality recalibration, and somatic mutation calling was performed using MuTect2. Real somatic mutations were retained after filtering out low-quality mutations and background noise, defining samples with at least one reported mutation as ctDNA-positive. The detailed methodological process was as previously reported (Lin et al., 2020, Ann Oncol (31), 517-524; Zhang et al., 2021, Nat Commun 12 (1):11).


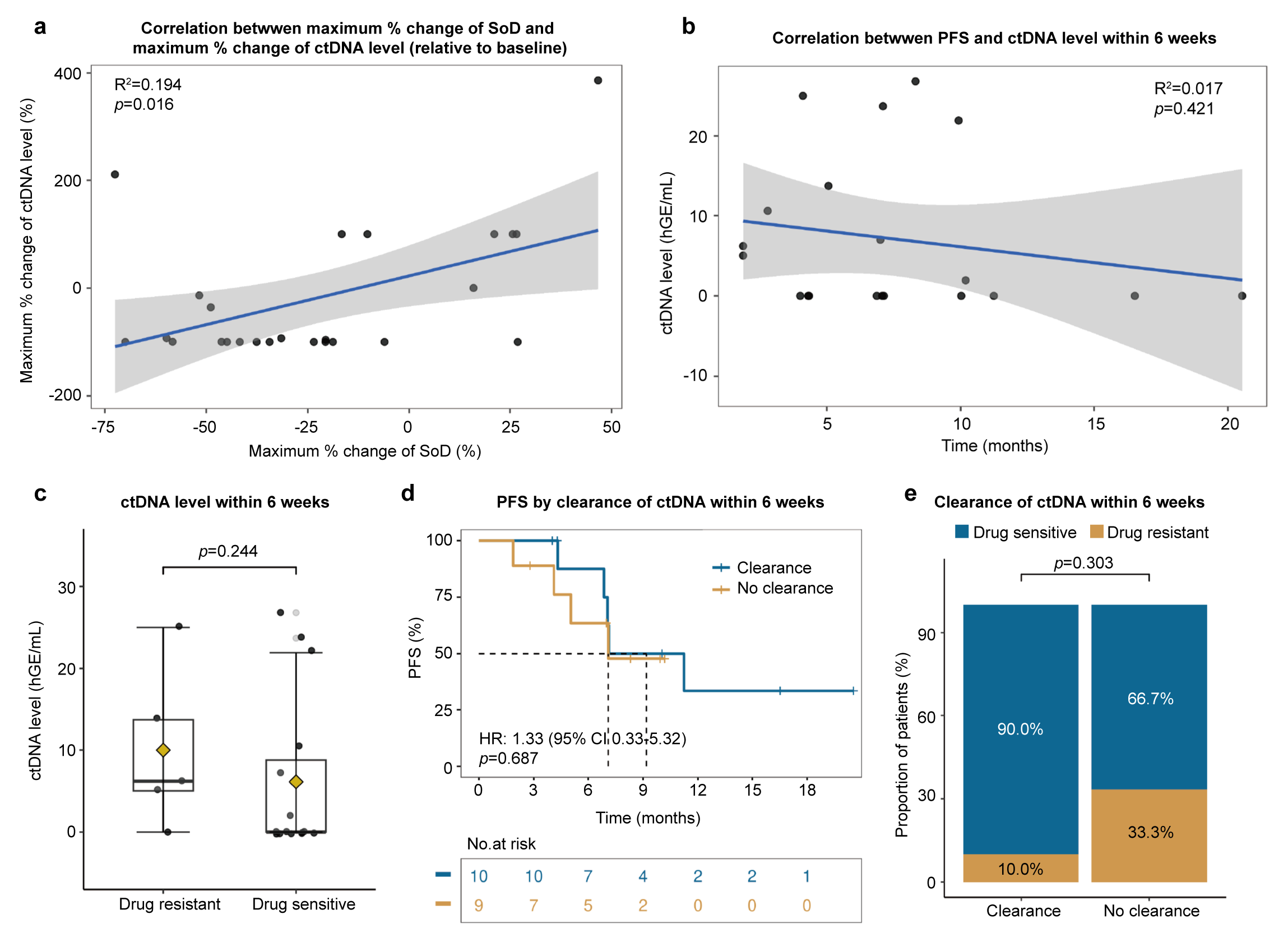


# Supplementary Figure 1. Biomarker analysis of ctDNA.

# (a) Linear correlation between maximum percent change in variant allele frequency and the SoD during dosing relative to baseline. (b) Linear correlation between ctDNA levels within 6 weeks of drug initiation and PFS time. (c) Distributional differences in ctDNA levels between drug-resistant and drug-sensitive patients within 6 weeks of drug initiation. (d) Kaplan-Meier plot demonstrating the prognostic difference between patients who showed ctDNA clearance within 6 weeks of drug initiation and those who did not. (e) Difference in the proportion of drug-resistant versus drug-sensitive patients who showed ctDNA clearance within 6 weeks of drug initiation versus those who did not. PFS, progression-free survival; SoD, sum of diameter.

# Supplementary Table 1. Summary of treatment-related serious adverse events per cohort

|  | **3.2 mg/kg**  **(n=3)** | **4.8 mg/kg**  **(n=43)** | **5.6 mg/kg**  **(n=11)** | **6.4 mg/kg**  **(n=3)** | **8.0 mg/kg**  **(n=3)** | **Total**  **(N=63)** |
| --- | --- | --- | --- | --- | --- | --- |
| Any event | 1 (33.3) | 5 (11.6) | 1 (9.1) | 3 (100) | 1 (33.3) | 11 (17.5) |
| Anaemia | 0 | 3 (7.0) | 0 | 0 | 0 | 3 (4.8) |
| Febrile neutropenia | 0 | 1 (2.3) | 0 | 0 | 1 (33.3) | 2 (3.2) |
| Interstitial lung disease | 0 | 0 | 0 | 2 (66.7) | 0 | 2 (3.2) |
| Neutrophil count decreased | 0 | 2 (4.7) | 0 | 0 | 1 (33.3) | 3 (4.8) |
| Platelet count decreased | 0 | 1 (2.3) | 0 | 0 | 1 (33.3) | 2 (3.2) |
| White blood cell count decreased | 0 | 2 (4.7) | 0 | 0 | 0 | 2 (3.2) |
| Asthenia | 0 | 1 (2.3) | 0 | 0 | 0 | 1 (1.6) |
| Bilirubin conjugated increased | 0 | 0 | 0 | 0 | 0 | 0 |
| Blood bilirubin increased | 0 | 0 | 0 | 0 | 0 | 0 |
| Pneumonia | 0 | 0 | 0 | 1 (33.3) | 0 | 1 (1.6) |
| Pulmonary tuberculosis | 0 | 0 | 0 | 0 | 1 (33.3) | 1 (1.6) |
| Stomatitis | 0 | 1 (2.3) | 0 | 0 | 0 | 1 (1.6) |
| Vomiting | 1 (33.3) | 1 (2.3) | 0 | 0 | 0 | 2 (3.2) |
| Hemoptysis | 0 | 0 | 1 (9.1) | 0 | 0 | 1 (1.6) |
| Nausea | 0 | 1 (2.3) | 0 | 0 | 0 | 1 (1.6) |

Data are n (%).

## Supplementary Table 2. Pharmacokinetic parameters after single dosing (cycle 1)

|  | | **3.2 mg/kg  (n=3)** | **4.8 mg/kg  (n=41)** | | **5.6 mg/kg  (n=10)** | | **6.4 mg/kg  (n=3)** | **8.0 mg/kg  (n=3)** | | |
| --- | --- | --- | --- | --- | --- | --- | --- | --- | --- | --- |
| SHR-A1811 | |  |  | |  | |  |  | | |
| C_max_ (μg/mL) | | 77 (9) | 106 (20) | | 133 (18) | | 164 (28) | 175 (23) | | |
| AUC_0-21d_ (day·μg/mL) | | 391 (38) | 592 (118) | | 746 (84.6) | | 739 (75.5) | 975 (248) | | |
| AUC_0-∞_ (day·μg/mL) | | 412 (39) | 659 (145) | | 845 (107) | | 829 (176) | 1170 (481) | | |
| AUC_0-t_ (day·μg/mL) | | 391 (38) | 588 (121) | | 728 (141) | | 750 (78) | 995 (252) | | |
| t_1/2_ (day) | | 5.1 (0.4) | 6.4 (1.0) | | 6.9 (1.0) | | 6.5 (3.4) | 7.5 (3.4) | | |
| T_max_ (day) | | 0.0688  (0.0639-0.0701) | 0.0632  (0.0590-0.3810) | | 0.1040  (0.0611- 0.1650) | | 0.0625  (0.0576-0.0625) | 0.0625  (0.0625-0.0625) | | |
| CL (L/day) | | 0.5 (0.1) | 0.5 (0.1) | | 0.4 (0.1) | | 0.6 (0.2) | 0.4 (0.2) | | |
| V_ss_ (L) | | 3.6 (0.4) | 4.0 (0.9) | | 3.8 (0.8) | | 4.4 (1.7) | 3.8 (0.4) | | |
| MRT (day) | | 7.2 (0.3) | 8.8 (1.4) | | 9.4 (1.3) | | 8.4 (4.3) | 10.1 (5.0) | | |
| Total antibody | |  |  | |  | |  |  | | |
| C_max_ (μg/mL) | | 72 (7) | 108 (20) | | 129 (17) | | 158 (29) | 172 (15) | | |
| AUC_0-21d_ (day·μg/mL) | | 367 (40) | 590 (118) | | 750 (85) | | 730 (64) | 950 (222) | | |
| AUC_0-∞_ (day·μg/mL) | | 388 (41) | 665 (151) | | 857 (112) | | 829 (177) | 1140 (451) | | |
| AUC_0-t_ (day·μg/mL) | | 367 (40) | 586 (123) | | 730 (140) | | 742 (67) | 969 (226) | | |
| t_1/2_ (day) | | 5.1 (0.3) | 6.7 (1.2) | | 7.2 (1.0) | | 6.6 (3.7) | 7.6 (3.6) | | |
| T_max_ (day) | | 0.0688  (0.0639- 0.0701) | 0.0632  (0.0583-0.1470) | | 0.0653  (0.0604-0.1470) | | 0.0625  (0.0625-0.1400) | 0.0625  (0.0625-0.1460) | | |
| CL (L/day) | | 0.5 (0.1) | 0.5 (0.1) | | 0.4 (0.1) | | 0.6 (0.2) | 0.4 (0.2) | | |
| V_ss_ (L) | | 3.9 (0.5) | 4.1 (0.9) | | 3.9 (0.7) | | 4.6 (1.8) | 4.0 (0.5) | | |
| MRT (day) | | 7.3 (0.2) | 9.1 (1.6) | | 9.7 (1.3) | | 8.9 (4.7) | 10.3 (5.1) | | |
| Free payload |  | |  | |  | |  |  | | |
| C_max_ (ng/mL) | 1.5 (0.2) | | | 2.6 (1.3) | 2.6 (0.6) | 4.8 (2.6) | | | 3.9 (1.0) |  |
| AUC_0-21d_ (day·ng/mL) | 8.6 (0.7) | | | 13.9 (5.5) | 15.0 (3.3) | 23.0 (6.5) | | | 23.5 (7.4) |  |
| AUC_0-∞_ (day·ng/mL) | 9.0 (0.8) | | | 14.8 (5.8) | 16.2 (3.5) | 24.4 (6.6) | | | 25.2 (7.6) |  |
| AUC_0-t_ (day·ng/mL) | 8.6 (0.7) | | | 13.9 (5.6) | 14.6 (4.0) | 23.3 (6.5) | | | 22.8 (6.2) |  |
| t_1/2_ (day) | 4.7 (0.0) | | | 5.2 (0.6) | 5.9 (1.1) | 5.3 (1.5) | | | 5.5 (1.1) |  |
| T_max_ (day) | 0.3980  (0.3960-0.4010) | | | 0.3960  (0.0236-1.0600) | 0.3990  (0.1470-1.0200) | 0.3960  (0.3890-0.3960) | | | 1.0600  (0.3960-1.0600) |  |
| MRT (day) | 6.7 (0.2) | | | 6.9 (0.8) | 7.6 (1.0) | 6.8 (1.9) | | | 7.6 (1.8) |  |

Data are mean (standard deviation) or median (range). AUC_0-∞_, area under the plasma-concentration curve from time zero to infinity; AUC_0-21d_, area under the plasma-concentration curve from time zero to day 21; AUC_0-t_, area under the plasma-concentration curve from time zero to the last measurable concentration; CL, clearance; C_max_, maximum concentration; MRT, mean residence time; t_1/2_, terminal elimination half-life; T_max_, time to C_max_; V_ss_, steady-state volume of distribution.

# Supplementary Table 3. Pharmacokinetic parameters after multiple dosing (cycle 3)*

|  | **3.2 mg/kg  (n=3)** | **4.8 mg/kg  (n=41)** | **5.6 mg/kg  (n=10)** | **6.4 mg/kg  (n=3)** |
| --- | --- | --- | --- | --- |
| SHR-A1811 |  |  |  |  |
| C_max_ (μg/mL) | 70 (6) | 120 (22) | 132 (19) | 160 (NC) |
| C_min_ (μg/mL) | 4.1 (0.2) | 10.2 (4.6) | 10.2 (2.4) | 6.9 (NC) |
| AUC_ss_ (day·μg/mL) | 472 (55) | 873 (182) | 969 (70) | 897 (NC) |
| AUC_0-∞_ (day·μg/mL) | 524 (50) | 1040 (267) | 1140 (105) | 945 (NC) |
| AUC_0-t_ (day·μg/mL) | 472 (55) | 887 (177) | 981 (118) | 897 (NC) |
| t_1/2_ (day) | 6.7 (0.8) | 8.1 (1.7) | 8.0 (0.7) | 4.8 (NC) |
| T_max_ (day) | 0.124 (0.115-0.132) | 0.0347 (0.0208-0.125) | 0.0354 (0.0333-0.106) | 0.0208 (0.0208-0.0208) |
| CL_ss_ (L/day) | 0.4 (0.0) | 0.4 (0.1) | 0.3 (0.1) | 0.4 (NC) |
| V_ss_ (L) | 3.8 (0.2) | 3.7 (0.8) | 3.7 (0.5) | 2.5 (NC) |
| MRT (day) | 8.7 (0.6) | 10.7 (2.1) | 10.7 (0.9) | 7.0 (NC) |
| R_ac,AUC_ | 1.2 (0.0) | 1.5 (0.3) | 1.3 (0.2) | 1.2 (NC) |
| Total antibody |  |  |  |  |
| C_max_ (μg/mL) | 70 (8) | 122 (19) | 136 (18) | 157 (NC) |
| C_min_ (μg/mL) | 3.9 (0.4) | 10.8 (5.1) | 10.7 (2.7) | 7.4 (NC) |
| AUC_ss_ (day·μg/mL) | 473 (47) | 891 (187) | 1020 (97) | 909 (NC) |
| AUC_0-∞_ (day·μg/mL) | 528 (41) | 1060 (277) | 1200 (105) | 962 (NC) |
| AUC_0-t_ (day·μg/mL) | 473 (47) | 901 (184) | 1030 (152) | 909 (NC) |
| t _½_ (day) | 6.8 (1.1) | 8.2 (1.7) | 8.0 (0.8) | 4.9 (NC) |
| T_max_ (day) | 0.1150 (0.0389-0.1320) | 0.1040 (0.0208-0.1250) | 0.0354 (0.0333-0.1060) | 0.0208 (0.0208-0.0208) |
| CL_ss_ (L/day) | 0.4 (0.0) | 0.3 (0.1) | 0.3 (0.1) | 0.4 (NC) |
| V_ss_ (L) | 3.9 (0.5) | 3.6 (0.7) | 3.5 (0.9) | 2.5 (NC) |
| MRT (day) | 8.9 (1.0) | 10.7 (2.1) | 10.6 (1.0) | 7.1 (NC) |
| R_ac,AUC_ | 1.3 (0.0) | 1.5 (0.3) | 1.4 (0.1) | 1.2 (NC) |
| Free payload |  |  |  |  |
| C_max_ (ng/mL) | 1.1 (0.1) | 1.6 (0.7) | 1.9 (0.6) | 2.5 (NC) |
| C_min_ (ng/mL) | 0.1 (0.0) | 0.1 (0.0) | 0.1 (0.1) | 0.1 (NC) |
| AUC_ss_ (day·ng/mL) | 6.8 (1.6) | 12.4 (3.3) | 13.7 (2.5) | 13.0 (NC) |
| AUC_0-∞_ (day·ng/mL) | 7.4 (1.6) | 13.9 (3.5) | 15.4 (3.1) | 13.8 (NC) |
| AUC_0-t_ (day·ng/mL) | 6.8 (1.6) | 12.5 (3.4) | 13.7 (2.2) | 13.0 (NC) |
| t_1/2_ (day) | 5.7 (0.5) | 6.3 (1.0) | 6.5 (1.6) | 5.6 (NC) |
| T_max_ (day) | 0.124 (0.1150-0.1320) | 0.104 (0.0806-7.0200) | 0.117 (0.1050-0.1190) | 0.105 (0.1050-0.1050) |
| MRT (day) | 7.6 (0.2) | 8.8 (1.5) | 9.2 (1.7) | 6.8 (NC) |
| R_ac,AUC_ | 0.8 (0.1) | 0.9 (0.1) | 0.9 (0.1) | 0.8 (NC) |

Data are mean (standard deviation) or median (range). * PK data are available for 1 patient in the 6.4 mg/kg cohort and none in the 8.0 mg/kg cohort. AUC_0-∞,_ AUC from time 0 extrapolated to infinity; AUC_0-t_, area under the plasma-concentration curve from time zero to the last measurable concentration; AUC_ss_, area under the plasma-concentration curve at steady state; CL_ss_, clearance at steady state; C_max_, maximum concentration; C_min_, minimum concentration; MRT, mean residence time; NC, not calculated; R_ac_, accumulation ratio; t_1/2_, terminal elimination half-life; T_max_, time to C_max_; V_ss_, steady-state volume of distribution.

# Supplementary Table 4. Efficacy by *HER2* mutation

| **Patient** | **Treatment** | ***HER2* mutation exon location** | ***HER2* mutation type** | **Amino Acid Change** | **HER2 expression (IHC)** | **Confirmed best overall response** |
| --- | --- | --- | --- | --- | --- | --- |
| 1 | 3.2mg/kg | 20 | Insertion | A775_G776insYVMA | N/A | SD |
| 2 | 3.2mg/kg | 20 | Insertion | P780_Y781insGSP | N/A | PR |
| 3 | 3.2mg/kg | 20 | Insertion | G776>VC | N/A | PR |
| 4 | 4.8mg/kg | 17 | SNV | V659E | N/A | PR |
| 5 | 4.8mg/kg | 17 | SNV | I655V | N/A | SD |
| 6 | 4.8mg/kg | 20 | Insertion | A775_G776insYVMA | N/A | SD |
| 7 | 4.8mg/kg | 20 | Insertion | A775_G776insYVMA | N/A | PR |
| 8 | 4.8mg/kg | 20 | Insertion | A775_G776insYVMA | 0 | PR |
| 9 | 4.8mg/kg | 20 | Insertion | A775_G776insYVMA | 0 | PR |
| 10 | 4.8mg/kg | 20 | Insertion | G776delinsAVGC | 1+ | PR |
| 11 | 4.8mg/kg | 20 | Insertion | A775_G776insSVMA | 0 | SD |
| 12 | 4.8mg/kg | 20 | Insertion | A775_G776insYVMA | 0 | PR |
| 13 | 4.8mg/kg | 20 | Insertion | A775_G776insYVMA | 2+ | PR |
| 14 | 4.8mg/kg | 20 | Insertion | P780_Y781insGSP | N/A | SD |
| 15 | 4.8mg/kg | 20 | Insertion | A775_G776insVVMA | N/A | SD |
| 16 | 4.8mg/kg | 20 | Insertion | G776>LC | N/A | SD |
| 17 | 4.8mg/kg | 20 | Insertion | A775_G776insYVMA | N/A | PR |
| 18 | 4.8mg/kg | 20 | Insertion | A775_G776insYVMA | 0 | PR |
| 19 | 4.8mg/kg | 20 | Insertion | A775_G776insYVMA | N/A | SD |
| 20 | 4.8mg/kg | 20 | Insertion | A775_G776insYVMA | 0 | SD |
| 21 | 4.8mg/kg | 20 | Insertion | V777delinsGAPL | N/A | PR |
| 22 | 4.8mg/kg | 20 | Insertion | A775_G776insYVMA | 0 | SD |
| 23 | 4.8mg/kg | 20 | Insertion | A775_G776insYVMA | 0 | SD |
| 24 | 4.8mg/kg | 20 | Insertion | G776>VC | N/A | SD |
| 25 | 4.8mg/kg | 20 | Insertion | P780_Y781insGSP | 1+ | SD |
| 26 | 4.8mg/kg | 20 | Insertion | A775_G776insYVMA | 0 | PR |
| 27 | 4.8mg/kg | 20 | Insertion | A775_G776insYVMA | N/A | PD |
| 28 | 4.8mg/kg | 20 | Insertion | A775_G776insYVMA | 0 | SD |
| 29 | 4.8mg/kg | 17 | SNV | V659D | 0 | SD |
| 30 | 4.8mg/kg | 20 | Insertion | A775_G776insYVMA | 0 | SD |
| 31 | 4.8mg/kg | 20 | Insertion | G776>VC | N/A | PR |
| 32 | 4.8mg/kg | 20 | Insertion | A775_G776insYVMA | 0 | PR |
| 33 | 4.8mg/kg | 20 | Insertion | A775_G776insYVMA | 0 | PR |
| 34 | 4.8mg/kg | 19 20 | SNV | D769Y V777L | 1+ | PR |
| 35 | 4.8mg/kg | 20 | Insertion | A775_G776insYVMA | 1+ | SD |
| 36 | 4.8mg/kg | 19 | SNV | L755S | 3+ | SD |
| 37 | 4.8mg/kg | 20 | Insertion | A775_G776insYVMA | N/A | SD |
| 38 | 4.8mg/kg | 24 | SNV | R966P | 0 | NE |
| 39 | 4.8mg/kg | 20 | Insertion | A775_G776insYVMA | 0 | SD |
| 40 | 4.8mg/kg | 20 | Insertion | N/A | 1+ | PR |
| 41 | 4.8mg/kg | 20 | Insertion | G776>VC | 1+ | SD |
| 42 | 4.8mg/kg | 20 | Insertion | A775_G776insYVMA | 1+ | PR |
| 43 | 4.8mg/kg | 20 | Insertion | P780_Y781insGSP | N/A | SD |
| 44 | 4.8mg/kg | 20 | Insertion | A775_G776insYVMA | 0 | PR |
| 45 | 4.8mg/kg | 20 | Insertion | A775_G776insYVMA | N/A | SD |
| 46 | 4.8mg/kg | 20 | Insertion | A775_G776insYVMA | N/A | SD |
| 47 | 5.6mg/kg | 20 | Insertion | A775_G776insYVMA | N/A | SD |
| 48 | 5.6mg/kg | 20 | Insertion | G776>VC | 1+ | SD |
| 49 | 5.6mg/kg | 20 | Insertion | P780_Y781insGSP | N/A | NE |
| 50 | 5.6mg/kg | 20 | Insertion | A775_G776insYVMA | 1+ | SD |
| 51 | 5.6mg/kg | 20 | Insertion | P780_Y781insGSP | N/A | SD |
| 52 | 5.6mg/kg | 20 | Insertion | N/A | N/A | SD |
| 53 | 5.6mg/kg | 20 | Insertion | A775_G776insYVMA | N/A | NE |
| 54 | 5.6mg/kg | 20 | Insertion | N/A | N/A | SD |
| 55 | 5.6mg/kg | 20 | Insertion | A775_G776insYVMA | 0 | PR |
| 56 | 5.6mg/kg | 20 | Insertion | G776>LC | 1+ | PD |
| 57 | 5.6mg/kg | 20 | Insertion | A775_G776insYVMA | N/A | SD |
| 58 | 6.4mg/kg | 20 | Insertion | A775_G776insYVMA | N/A | NE |
| 59 | 6.4mg/kg | 20 | Insertion | A775_G776insYVMA | N/A | PR |
| 60 | 6.4mg/kg | 20 | Insertion | A775_G776insYVMA | N/A | SD |
| 61 | 8.0mg/kg | 20 | Insertion | N/A | N/A | PR |
| 62 | 8.0mg/kg | 20 | Insertion | A771_Y772insAYVM | N/A | PR |
| 63 | 8.0mg/kg | 19 20 | SNV | D769Y V777L | N/A | SD |

IHC, immunohistochemistry; N/A, not available; NE, not evaluable; PR, partial response; SD, stable disease; SNV, single-nucleotide polymorphism.

# Supplementary Table 5. Efficacy by location of *HER2* mutation

|  | ***HER2* mutation** | |
| --- | --- | --- |
|  | **Non-kinase domain^a^ (n=3)** | **Kinase domain**  **(n=60)** |
| Best overall response, n (%) | |  |
| Complete response | 0 | 0 |
| Partial response | 1 (33.3) | 23 (38.3) |
| Stable disease | 2 (66.6) | 31 (51.7) |
| Progressive disease | 0 | 2 (3.3) |
| Not evaluable | 0 | 4 (6.7) |
| ORR,% | 33.3 | 38.3 |
| DCR,% | 100 | 90.0 |

^a^ All single-nucleotide variant in exon 17. DCR, disease control rate; ORR, objective response rate.

# Supplementary Table 6. Sampling nodes for biomarker analysis

| **Patient** | **Sampling times** | **Sampling nodes** | **Sampling at baseline** | **Sampling**  **at EOT** |
| --- | --- | --- | --- | --- |
| 1 | 6 | C1, C5, C9, C17, C21, EOT | Yes | Yes |
| 2 | 2 | C1, C3 | Yes | No |
| 3 | 6 | C3, C9, C17, C23, C25, C27 | No | No |
| 4 | 3 | C1, C7, C10 | Yes | No |
| 5 | 3 | C1, C4, EOT | Yes | Yes |
| 6 | 7 | C1, C3, C7, C11, C13, C15, C18 | Yes | No |
| 7 | 2 | C1, EOT | Yes | Yes |
| 8 | 5 | C1, C3, C7, C15, C22 | Yes | No |
| 9 | 3 | C1, C2, C5 | Yes | No |
| 10 | 6 | C1, C4, C10, C13, C17, C22 | Yes | No |
| 11 | 4 | C1, C3, C7, EOT | Yes | Yes |
| 12 | 3 | C1, C3, C7 | Yes | No |
| 13 | 3 | C1, C3, C6 | Yes | No |
| 14 | 2 | C2, C3 | No | No |
| 15 | 4 | C1, C3, C6, EOT | Yes | Yes |
| 16 | 7 | C1, C4, C5, C9, C13, C15, EOT | Yes | Yes |
| 17 | 3 | C1, C4, C6 | Yes | No |
| 18 | 5 | C1, C5, C9, C17, C18 | Yes | No |
| 19 | 1 | C1 | Yes | No |
| 20 | 4 | C1, C3, C8, C11 | Yes | No |
| 21 | 2 | C1, C3 | Yes | No |
| 22 | 3 | C1, C3, C4 | Yes | No |
| 23 | 3 | C1, C3, C4 | Yes | No |
| 24 | 3 | C1, C3, C8 | Yes | No |
| 25 | 6 | C1, C3, C5, C11, C13, C14 | Yes | No |
| 26 | 4 | C1, C3, C11, C14 | Yes | No |
| 27 | 3 | C1, C3, C8 | Yes | No |
| 28 | 4 | C1, C3, C8, C9 | Yes | No |

Samples were collected from 28 consecutive patients at a single study center. In this patient subset, the objective response rate and disease control rate were 46.4% and 96.4%, respectively, and the median progression-free survival was 7.1 months. A total of 107 blood samples were collected for next-generation sequencing. Baseline blood samples were collected before drug dosing, and blood samples during drug administration were collected on the first day of each dose cycle (optionally). C, cycle; EOT, end of treatment.
